# Resignations, Appointments and Deaths in Medical Schools

**Published:** 1841-05

**Authors:** 


### RESIGNATIONS, APPOINTMENTS AND DEATHS IN MEDICAL SCHOOLS.

Change is a characteristic feature of our Medical Schools. Very lately several professorships have been vacated and most of them filled by new incumbents.

Our elder sister of the West, Transylvania, has lost Professor Smith by resignation and return to Baltimore—his chair not yet filled, but an election expected to take place in June. The late Professor, it is said, will co-operate in the reorganization of the University of Maryland. The Jefferson Medical College has experienced still greater changes. In the month of February, she lost, by death, the amiable and respected Dr. Green, Professor of Chemistry, one of the original members of the Faculty. Subsequently Professors Pattison and Revere resigned the chairs of Anatomy and the Theory and Practice, and accepted appointments in the University of the *City* of New York. These events were followed by a kind of reorganization of the former institution, and the appointment of several gentlemen, who have not hitherto belonged to incorporated medical schools. Dr. Pancoast being transferred to Anatomy, Dr. Mutter was appointed Professor of the Institutes of Surgery, and Dr. Randolph of Operative Surgery; Dr. Huston was transferred from Obstetrics to Materia Medica, and Dr. Meigs elected his successor, Dr. Dunglison being placed in the chair of Institutes; Dr. Mitchell was chosen to that of Theory and Practice, and Dr. Bache that of Chemistry. The Pennsylvania College about the same time lost her Professor of Materia Medica, Dr. Calhoun, and his place has not been supplied. D.

